# A Surfactant-Induced Functional Modulation of a Global Virulence Regulator from *Staphylococcus aureus*

**DOI:** 10.1371/journal.pone.0151426

**Published:** 2016-03-18

**Authors:** Sukhendu Mandal, Avisek Mahapa, Anindya Biswas, Biswanath Jana, Soumitra Polley, Keya Sau, Subrata Sau

**Affiliations:** 1 Department of Biochemistry, Bose Institute, Kolkata, West Bengal, India; 2 Department of Biotechnology, Haldia Institute of Technology, Haldia, West Bengal, India; National University of Singapore, SINGAPORE

## Abstract

Triton X-100 (TX-100), a useful non-ionic surfactant, reduced the methicillin resistance in *Staphylococcus* aureus significantly. Many *S*. *aureus* proteins were expressed in the presence of TX-100. SarA, one of the TX-100-induced proteins, acts as a global virulence regulator in *S*. *aureus*. To understand the effects of TX-100 on the structure, and function of SarA, a recombinant *S*. *aureus* SarA (rSarA) and its derivative (C9W) have been investigated in the presence of varying concentrations of this surfactant using various probes. Our data have revealed that both rSarA and C9W bind to the cognate DNA with nearly similar affinity in the absence of TX-100. Interestingly, their DNA binding activities have been significantly increased in the presence of pre-micellar concentration of TX-100. The increase of TX-100 concentrations to micellar or post-micellar concentration did not greatly enhance their activities further. TX-100 molecules have altered the secondary and tertiary structures of both proteins to some extents. Size of the rSarA-TX-100 complex appears to be intermediate to those of rSarA and TX-100. Additional analyses show a relatively moderate interaction between C9W and TX-100. Binding of TX-100 to C9W has, however, occurred by a cooperative pathway particularly at micellar and higher concentrations of this surfactant. Taken together, TX-100-induced structural alteration of rSarA and C9W might be responsible for their increased DNA binding activity. As TX-100 has stabilized the somewhat weaker SarA-DNA complex effectively, it could be used to study its structure in the future.

## Introduction

*Staphylococcus aureus*, a Gram-positive bacterium, encodes various virulence factors (such as enterotoxins, α-hemolysin, exfoliative toxins, protein A, β-hemolysin, clumping factor, δ-hemolysin, fibronectin-binding protein, phenol-soluble modulins, iron-uptake protein, proteases, capsule, lipases, biofilm, nucleases, Panton-Valentine leukocidin, peptidoglycan hydrolases, etc.) and virulence regulators (namely, *agr*, *sarA*, *saeRS*, *codY*, *sigB*, etc.) for causing diseases (e.g., boils, carbuncles, abscesses, pneumonia, endocarditis, osteomyelitis, sepsis, etc.) in human and other animals [1–6]. Expression of the majority of the virulence factors in *S*. *aureus* appeared to be dependently or independently regulated by *agr* and *sarA*, two global virulence regulators in *S*. *aureus* [1–6].

SarA, a *sarA*-encoded protein, possesses 124 amino acid residues, forms dimers in solution, carries a flexible C-terminal end and is composed of α-helix [1, 7, 8]. Interestingly, *S*. *aureus* encodes ten SarA paralogs such as MgrA, Rot, SarR to SarV, SarX, SarY, and SarZ [5, 6, 9]. The Sar family members in *S*. *aureus* were reported to control the expression of various genes including the genes involved in the virulence. The three-dimensional structures of several Sar family of proteins (such as SarA, SarR, SarS, SarZ, Rot, and MgrA) were solved and found to possess a winged-helix conformation [9–15]. The winged-helix structure of SarA in particular is composed of two globular monomers [12]. There are five α-helices (α1-α5), three β-strands (β1-β3), and multiple loops in each SarA monomer. While α1, α2, and α5 are involved in SarA dimerization, α3, α4, β2, and β3 were speculated to be involved in the DNA binding. The helices α3 and α4 appear to form a helix-turn-helix (HTH) motif, whereas, β2 and β3 produce a β-hairpin or winged region in SarA [12].

SarA has exhibited binding to the promoters of many virulence-associated genes/loci such as *spa*, *hla*, *cna*, *fnbA*, *ica*, *tst*, *bap*, *agr*, *rot*, *sarS*, *sarV*, etc. [1–6]. It has also shown binding to the promoters of the genes encoding thioredoxin reductase and superoxide dismutase, which usually protect *S*. *aureus* from the reactive oxygen species [16, 17]. Interestingly, SarA acts not only as a repressor but also as an activator [1–6, 16, 17]. While the binding of SarA to the *cna*, *rot*, *sod*, *trxB*, *sarV*, and *spa* promoters inhibited transcription, that to the *hla*, *ica*, *tst*, *bap*, *fnbA*, and *agr* promoters augmented the transcription of the linked genes. In addition, SarA has also repressed its own production by binding to *P1* and *P3* promoters [9]. Microarray analyses have suggested that SarA is involved not only in the pathogenesis but also in the diverse cellular activities including metabolism, and transport [18]. In addition, SarA has also shown binding to various mRNA species, indicating its roles in the regulation of the gene expression at the post-transcriptional level [19].

*S*. *aureus* becomes one of the dreaded pathogens today primarily due to the non-availability of vaccine and the emergence and dissemination of *S*. *aureus* strains, which are resistant to multiple antibiotics [1–6]. To exterminate such strains, novel antistaphylococcal compounds capable of disrupting the SarA-DNA complex may be useful [6, 20]. Structure of the SarA-promoter DNA complex with the potentiality in the drug discovery is not known. Such a structure could be determined easily if SarA binds to the cognate DNA with high affinity. The DNA binding affinity of SarA *in vitro* appeared to be somewhat weak and was different in different buffer [1, 2, 5, 6]. Buffers with low pH or containing high concentration of any reducing agent have enhanced the binding affinity of SarA marginally [21]. The phosphorylation-dephosphorylation status of SarA has also modulated its binding activity to some extent [22, 23]. By analyzing the compositions of different SarA binding buffers [9, 20–22], we have recently learnt that the DNA binding affinity of SarA is relatively higher in the buffers containing micellar concentration of Triton X-100 (TX-100), a non-ionic surfactant [24]. Numerous membrane proteins were purified using this detergent [25–26]. Structures and function of many non-membrane proteins were also modulated by TX-100 as well [27–29]. Interestingly, TX-100 reduced the methicillin resistance in various *S*. *aureus* strains remarkably [30–33]. A proteomics study has even revealed the increased expression of SarA in the presence of TX-100 [34]. Thus far, no systematic study has been carried out to precisely understand the effects of TX-100 on the structure and function of SarA. In the present study, we have investigated the effects of TX-100 on the structure and function of a recombinant SarA (rSarA) and its mutant (C9W) by various *in vitro* methods. Mutant C9W carries a Cys to Trp substitution at position 9 of rSarA [8]. Our data have demonstrated a significant increase of the DNA binding activity of both rSarA and C9W in the presence of pre-micellar, micellar, and post-micellar concentrations of TX-100. In addition, TX-100 micelles have marginally altered the structures of these proteins and formed complexes with them. Binding of TX-100 micelles to C9W has occurred by a cooperative manner.

## Materials and Methods

### Materials

Acrylamide, bis-acrylamide, TX-100, glutaraldehyde, PMSF (phenylmethane sulfonylfluoride), urea, and IPTG (isopropyl β-D-thiogalactopyranoside) were bought from Merck, SRL, or Sigma. All other reagents were of the highest purity available. Oligonucleotides, polymerase chain reaction (PCR) kit, plasmid DNA purification kit, the gel extraction kit, restriction and modifying enzymes, DNA and protein markers, alkaline phosphatase-linked goat anti-mouse antibody, anti-His antibody, and Ni-NTA resin were purchased from Genetix Biotech Asia Pvt Ltd., GE Healthcare Biosciences Ltd., Fermentas, Hysel India Pvt Ltd., Santa cruz Biotechnology Inc., and Qiagen. Radioactive nucleotide [γ-^32^P] ATP was procured from the Bhabha Atomic Research Center. *S*. *aureus* strain Newman was obtained as a generous gift from Prof. Chia Y Lee, University of Arkansas for Medical Sciences. *E*. *coli* BL21(DE3) and plasmid pET28a were presented by the late Prof. P. Roy, Bose Institute. Oligonucleotides used in the present study were listed in S1 Table. rSarA was purified as described [8].

### Basic molecular biological techniques

All of the molecular biological methods such as plasmid DNA isolation, polymerase chain reaction (PCR), restriction enzyme digestion, agarose gel electrophoresis, labelling of DNA fragment by [γ-^32^P] ATP, DNA ligation, competent *E*. *coli* cell preparation, DNA transformation, estimation of protein and DNA, sodium dodecyl sulphate-polyacrylamide gel electrophoresis (SDS-PAGE), staining of polyacrylamide gel, Western blotting, native polyacrylamide gel electrophoresis, isolation of genomic DNA from *S*. *aureus* Newman, and sequencing of DNA fragments were performed as reported earlier [8, 35–39].

### Purification of C9W

To purify C9W, a PCR amplification of plasmid p1311 DNA [8] was performed using primers SarAC9W1 and SarAC9W2 (S1 Table) by a standard procedure [40]. The resulting DNA fragments were cloned to pET28a, an *E*. *coli*-specific expression vector. One of the yielded plasmids that carried the desired sequence was considered and designated p1336. *E*. *coli* SAU1336 was constructed by transforming *E*. *coli* BL21(DE3) with p1336. C9W was purified from SAU1336 cells by a standard affinity chromatography [8]. In brief, the IPTG-induced SAU1336 cells in buffer A [20 mM Tris-HCl (pH 8.0), 300 mM NaCl, 10 mM imidazole, 5% glycerol and 10 μg/ml PMSF] were broken by sonication. After removal of cell debris, the crude extract was subjected to a Ni-NTA column chromatography. Different fractions collected from the chromatography were analyzed by a SDS-13.5% PAGE (S1 Fig). The gel picture shows that the elution fraction primarily contains a protein with the molecular mass of ~16 kDa. The eluted protein could be C9W as it has shown significant binding to a ^32^P-labeled hla DNA [8] and yielded an intrinsic Trp fluorescence spectrum (see below). We dialyzed the eluted C9W against buffer B [20 mM phosphate buffer (pH 8.0), 100 mM NaCl, and 5% glycerol] or buffer C (buffer B containing distinct concentration of TX-100) for 12–16 h at 4°C. The molar concentration of rSarA or C9W was determined using the molecular mass of its monomer.

### Gel shift assay

To determine the DNA binding activities of rSarA and C9W, we carried out separate gel shift assays by a standard procedure with modifications [41]. Briefly, reaction mixtures (in buffer B or buffer C) containing varying amounts of protein and 0.1 nM ^32^P-labelled hla [8] or spa DNA (produced using Newman DNA and oligonucleotides Spa1 and Spa2; S1 Table) were incubated for 20 min on ice followed by the analysis of all samples by the 6% native PAGE. The amounts of DNA bound by rSarA or C9W were determined using the scanned (band intensity) data from the autoradiograms. The apparent equilibrium dissociation constant (*K*_d_) for the protein-DNA interaction was calculated by fitting the gel shift assay data to a sigmoid curve equation using Microcal Origin (Version 6.0).

### DNase I footprinting assay

To identify the binding site of rSarA, a DNase I footprinting assay was performed by a standard method [37, 42] with some modifications. Briefly, the 5’ end of hla2 DNA (S1 Table) was labeled with ^32^P using [γ-^32^P] ATP and T4 polynucleotide kinase [37]. A ^32^P-labeled DNA fragment was made by PCR amplification of *S*. *aureus* Newman DNA using labeled primer hla2 and unlabeled primer hla1 (S1 Table). Nearly 50 nM labeled hla DNA was incubated with 500 nM rSarA in buffer C (containing 0.7 mM Triton X-100 and 1 mM MgCl_2_) for 20 min on ice. The reaction mixture was treated with 0.5 units of DNase I for 15 min at room temperature. After termination of the reaction by a Stop solution, the chopped DNA fragments in the mixture were successively purified by the phenol-chloroform (1:1) extraction, and alcohol precipitation steps [37, 42]. To make control DNA fragments, the labeled hla DNA in the absence of rSarA was similarly digested with DNase 1. The adenosine + guanine and guanine sequencing ladders were produced using the labeled hla DNA by a standard method [37]. All of the DNA pieces were analyzed by a urea-8% PAGE as described [42]. The dried gel was analyzed by a phosphorimager to see the resolved DNA fragments [41].

### Shape and Size of rSarA

To determine the oligomeric status of rSarA, a glutaraldehyde-mediated cross-linking experiment of rSarA (10 μM) in the buffer B or buffer C was carried out as stated [38].

To determine the hydrodynamic radius of rSarA in the presence/absence of TX-100, dynamic light scattering (DLS) experiment of rSarA in buffer B or buffer C was performed by a standard method with some modifications [8, 43]. Briefly, buffer C with/without rSarA was centrifuged at 12000 rpm for 30 min followed by its filtration through 0.22 μm Millex syringe filter. rSarA in buffer B was also treated similarly. DLS experiments of the resulting protein (30 μM) and buffers were performed at a scattering angle of 90° using Zetasizer Nano S from Malvern Instruments. The hydrodynamic radii of rSarA, TX-100, and rSarA-TX-100 complex have been determined by fitting the yielded data to the Stokes-Einstein equation (Eq 1) using software installed in the scattering instrument.

$$R_{h}=kT/6\pi \eta D$$ (1)

where *R*_h_, k, T, *η*, and *D* are hydrodynamic radius, Boltzmann’s constant, absolute temperature, viscosity of the buffer, and translational diffusion coefficient, respectively.

### CD and fluorescence spectroscopy

To obtain clues about the secondary structures of rSarA and C9W, far-UV Circular Dichroism (CD) spectra (200–260 nm) of these macromolecules (10 μM) in buffer B or buffer C were recorded as mentioned earlier [38, 44]. To determine the quantity of different secondary structural elements in these proteins, their CD spectra were analyzed by CDNN [45]. To obtain clues about the tertiary structure of rSarA, near-UV CD spectrum (250–350 nm) of this protein (30 μM) in buffer B or buffer C was recorded as described [38, 44].

To know about the tertiary structure of C9W, the intrinsic Trp fluorescence spectrum (λ_ex_ = 295 nm and λ_em_ = 300–400 nm) of this rSarA derivative (2.5 μM) in buffer B or buffer C was recorded as demonstrated [38, 46]. The intrinsic fluorescence spectra of C9W, pre-equilibrated with 0–1.54 mM TX-100 for 20 min on ice, were also recorded as above. Using the intrinsic Trp fluorescence intensity values (at 336 nm) of 0–1.54 mM TX-100-equilibrated C9W, the average number of TX-100 molecule bound per C9W molecule (ν) was determined using a standard equation (Eq 2) as stated earlier [27].

ν=θ[TX−100]/[C9W] (2)

where *θ*, [*TX-100*], and [*C9W*] indicate the fraction of C9W bound to TX-100, total concentration of TX-100, and total concentration of C9W, respectively.

The fraction of C9W bound to TX-100 (*θ*) was calculated from the following equation [27]:

$$\theta =(I_{obs}-I_{free})/(I_{min}-I_{free})$$ (3)

where *I*_obs_, *I*_min_, and *I*_free_ denote Trp fluorescence intensity at any TX-100 concentration, Trp fluorescence intensity in the presence of TX-100 concentrations yielding saturation binding, and Trp fluorescence intensity in the absence of TX-100, respectively.

To determine the binding affinity of TX-100 to C9W, the Trp fluorescence intensity values obtained in the presence of 0–1.54 mM TX-100 were analyzed by the Scatchard equation (Eq 3) as described [27, 47].

r/c=Kn−Kr (4)

where *K*, *r*, *c*, and *n* indicate the binding affinity constant, moles of TX-100 bound per mole of C9W, unbound TX-100 concentration, and number of TX-100 binding sites on C9W, respectively. The unbound TX-100 concentration and bound TX-100 concentration were determined using the equations [*TX-100*](1-*θ*) and [*TX-100*]*θ*, respectively [27]. The Scatchard plot has been made by plotting *r/c* values against *r* values [47].

### Statistical analysis

All of the results were provided here as the means of at least three separate experiments with the standard deviation. Mean, standard deviation, and *p* values were estimated using the corresponding default statistical functions from Microsoft Excel. The two results were deemed noteworthy when the related *p* value was <0.05.

## Results

### Effects of TX-100 on the DNA binding affinity of rSarA

**Fig 1:**
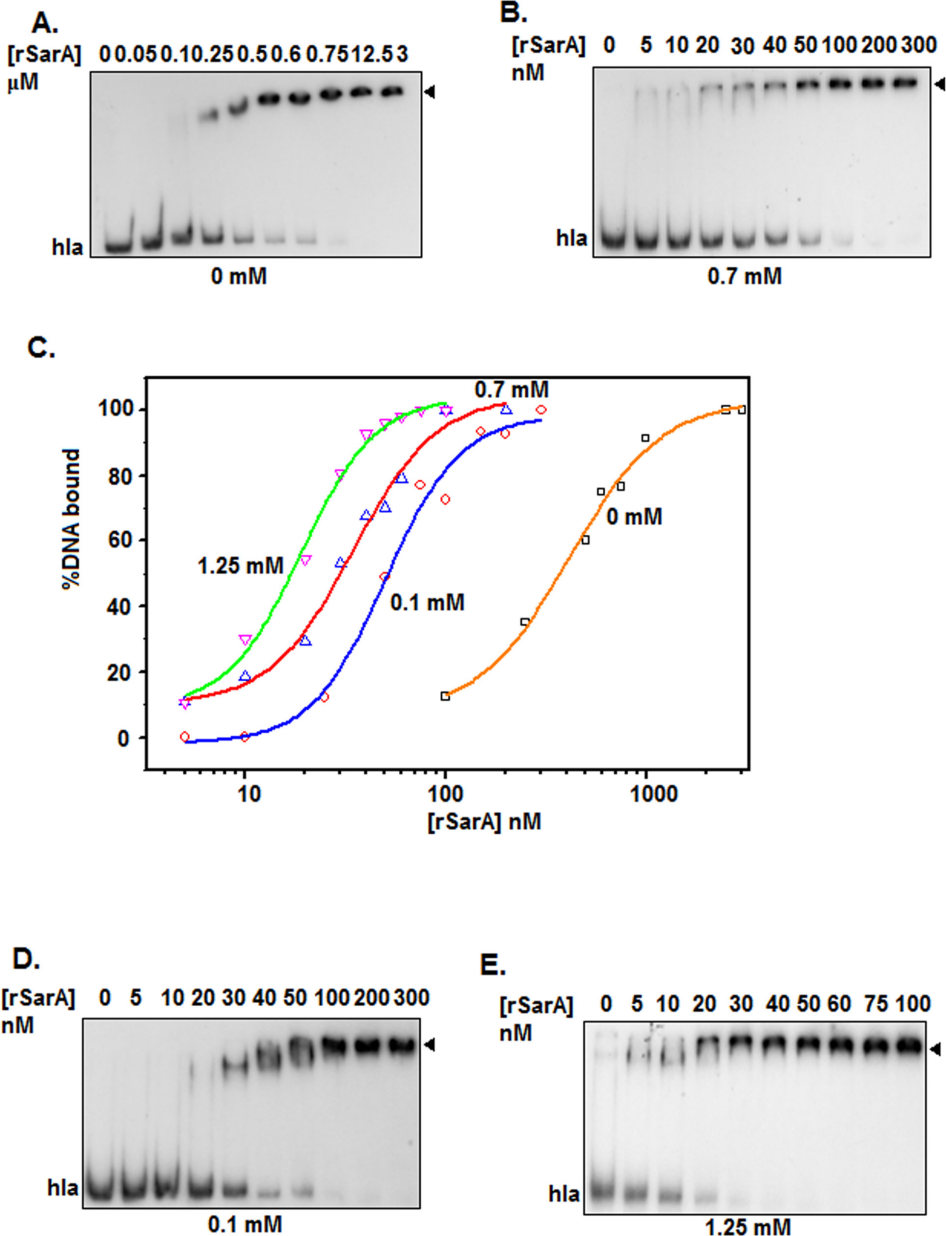
Gel shift assay in the presence of varying concentrations of TX-100. Autoradiograms show the equilibrium binding of rSarA to the ^32^P-labeled hla DNA in the presence of 0 mM (A), 0.7 mM (B), 0.1 mM (D), and 1.25 mM (E) TX-100. Arrowhead indicates the rSarA-hla DNA complex. All of the assays are performed at least three times. A set of autoradiograms are shown here. (C) rSarA DNA binding affinity. The amounts of rSarA bound to hla DNA in the presence of indicated concentrations of TX-100 are determined (using the data from the above autoradiograms) and plotted against the corresponding rSarA concentrations.

The critical micelle concentration of TX-100 in the aqueous solution ranges from ~0.2 to 0.9 mM [24]. To verify whether the micellar concentration of TX-100 truly enhances the DNA binding affinity of rSarA, we have studied the equilibrium binding of rSarA to ^32^P-labeled hla DNA in the presence of 0 and 0.7 mM TX-100 using separate gel shift assay. As reported previously [9, 20–22], the concentration of rSarA that started forming the rSarA-hla DNA complex in the absence of TX-100 (Fig 1A) was higher than that initiated the same in the presence of TX-100 micelles (Fig 1B). The plots of the percent DNA bound versus the rSarA concentrations, developed using the data from the autoradiograms in Fig 1A and 1B, have revealed that the apparent equilibrium dissociation constants (i.e. rSarA concentrations yielding 50% saturation of the input hla DNA) in the presence and absence of TX-100 are 35±4 and 364±8 nM, respectively (Fig 1C; Table 1). The hla DNA binding affinity of rSarA has, therefore, increased about ~950% in the presence of 0.7 mM TX-100 (*p* = 0.002).

**Table 1:** Effect of TX-100 on the DNA binding activity of protein.

Concentration of TX-100 (mM)	*K*_d_ values^a^ for hla DNA-rSarA interaction (nM)	*K*_d_ values^a^ for spa DNA-rSarA interaction (nM)	*K*_d_ values^a^ for hla DNA-C9W interaction (nM)
0	364±8	838±12	398±3
0.1	50±6		97±4
0.7	35±4	107±5	58±3
1.25	20±1		60±1

^a^The *K*_d_ values for the indicated protein-DNA interaction in the presence of different concentrations of TX-100 are determined from the autoradiograms and the resulting plots of different gel shift assays.

To determine whether the pre-micellar and post-micellar concentrations of TX-100 also modulate the DNA binding activity of rSarA, we have performed gel shift assays using the ^32^P-labeled hla DNA and rSarA in the presence of 0.1 and 1.25 mM TX-100, respectively. The rSarA-hla DNA complex is again formed at a rSarA concentration which is lower in the presence of TX-100 (pre-micellar and post-micellar) than in the absence of TX-100 (Fig 1D and 1E). The *K*_d_ values are determined from the resulting plots of the percent DNA bound versus the rSarA concentrations (Fig 1C and Table 1). There are about ~600% and ~1700% increase of the hla DNA binding affinity of rSarA in the presence of 0.1 mM, and 1.25 mM TX-100, respectively. Additional analysis, however, reveals no significant difference between the *K*_d_ values obtained at 0.7 mM and 0.1 mM or 1.25 mM TX-100 (all *p* values greater than 0.05).

To verify if the TX-100-mediated increase of the binding affinity of rSarA is DNA specific, we have studied the equilibrium binding of rSarA to ^32^P-labeled spa DNA in the presence of 0 and 0.7 mM concentrations of this detergent. As noticed with hla DNA (mentioned above), rSarA also shows comparatively higher binding affinity to spa DNA in the presence of 0.7 mM TX-100 (S2 Fig). The *K*_d_ values estimated from the plots of equilibrium binding of rSarA to spa DNA (S2C Fig) reveal that there is about eight times increase of the spa DNA binding activity of rSarA in the presence of 0.7 mM TX-100. The data indicate that the binding affinities of rSarA to both the hla and spa DNAs have been notably boosted byTX-100.

**Fig 2:**
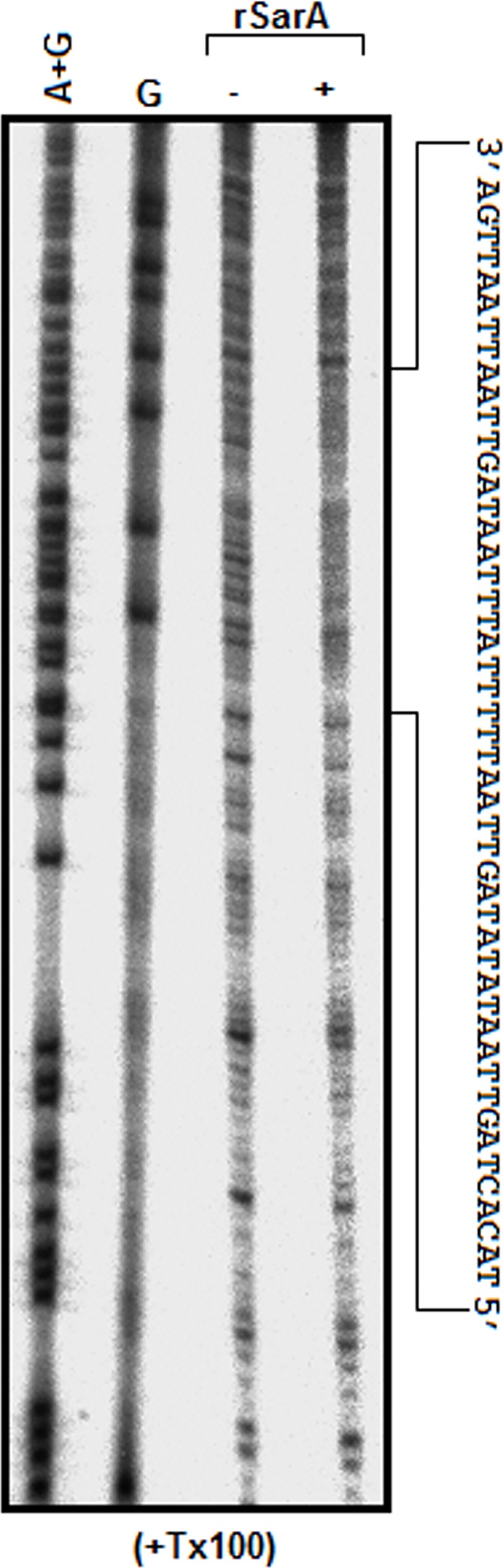
Autoradiogram of DNase I footprinting assay. The bottom strand labelled (with ^32^P) hla DNA was incubated with (+)/without (-) the saturating amount of rSarA followed by the digestion of the DNA with DNase I. The resulting DNA fragments, along with the G and A+G markers (made from the same labelled hla DNA by a standard method) were separated by a urea-8% PAGE. Sequence of the hla DNA protected by rSarA is shown at the right side of autoradiogram.

To determine whether TX-100 alters the rSarA binding location in the promoter DNA, we carried out a DNase I footprinting experiment using a saturating amount of rSarA and ^32^P-labeled hla DNA in the buffer containing 0.7 mM TX-100. Analysis of the footprint (Fig 2) indicates that the DNA binding location of rSarA in the presence of TX-100 remains nearly identical as reported for a recombinant SarA in the absence of this detergent [36].

### Effects of TX-100 on the secondary and tertiary structures of rSarA

**Fig 3:**
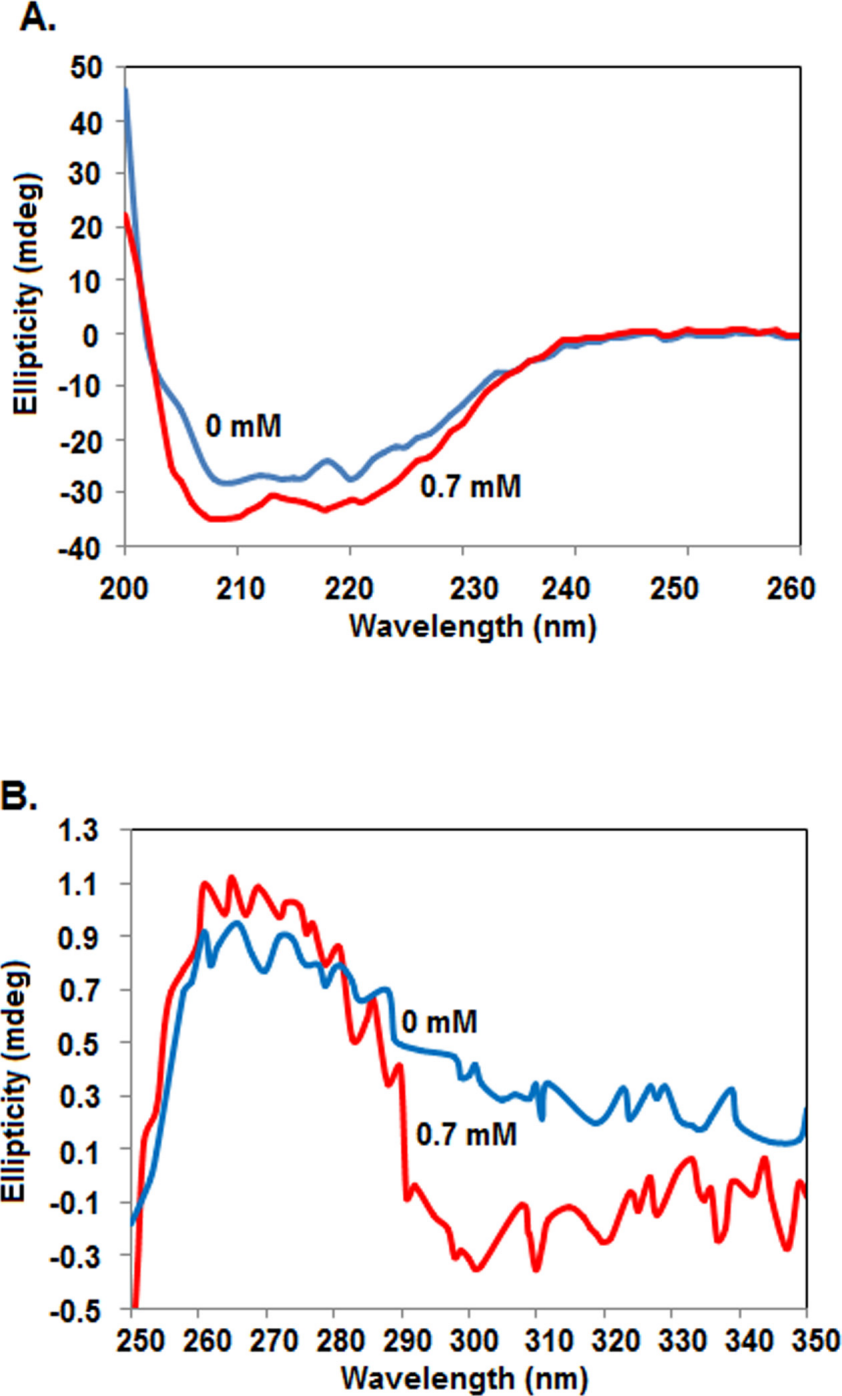
Secondary and tertiary structures of rSarA. Far-UV (A) and near-UV (B) CD spectra of rSarA in the presence of indicated concentrations of TX-100. All of the spectra are recorded at least three times. One set of spectra are shown here.

To see if TX-100 alters both the secondary and tertiary structures of rSarA, we have separately recorded the far- and near-UV CD spectra of this protein in the presence of 0 and 0.7 mM TX-100. To notice sufficient extent of structural alteration and to minimize noise, we have used 0.7 mM TX-100 in these spectroscopic studies. Fig 3A shows that far-UV CD spectrum of rSarA in the presence of 0.7 mM TX-100 is fairly different from its spectrum recorded in the absence of this detergent. Both the spectra are, however, composed of the peaks at ~208 and ~220 nm, indicating that rSarA carries varying extent of α-helix in the presence of 0 and 0.7 mM TX-100. Analysis of the spectra by CDNN [44] indicates that the contents of secondary structural elements (including α-helix) in rSarA have been altered in the presence of TX-100 (S2 Table).

The near-UV CD spectra of rSarA recorded in the presence of 0 and 0.7 mM TX-100 have shown a flattened peak of large positive ellipticity at around 260–285 nm (Fig 3B). Tyr residue usually produces peak at ~275–282 nm, whereas, Phe residue yields peak at ~255–270 nm [39, 44]. The peak at ~260–285 nm could be, therefore, due to the presence of six Tyr and four Phe residues in rSarA. The non-overlapping of the spectra, however, indicate the TX-100-induced alteration of tertiary structure of rSarA. The factors responsible for affecting the near-UV CD spectrum of any protein molecule are the number of aromatic amino acid residues, interaction among the neighboring aromatic amino acid residues, hydrogen bond, protein rigidity, polar groups, etcetera [39, 44]. Currently, the determinants those have partly changed the three-dimensional structure of rSarA in the presence of 0.7 mM TX-100 are not clearly known.

### Effects of TX-100 on the shape and size of rSarA

The shape and size of rSarA, like its structure, may be altered by TX-100 as well. To verify this hypothesis, we have performed various *in vitro* experiments with rSarA in the buffers containing 0 and 0.7 mM TX-100. The glutaraldehyde-mediated cross-linking experiment shows the formation of dimeric rSarA both in the presence and absence of TX-100 (S3A Fig). The gel filtration chromatography of rSarA in the 0 and 0.7 mM TX-100 containing buffers yielded primarily single peaks with the retention volumes of ~87.87 and ~87.6 ml, respectively (S3B Fig). Using the elution volumes of some monomeric proteins (data not shown) and that of TX-100-untreated rSarA, the apparent molecular mass of rSarA in the absence of TX-100 was calculated to be ~31.95 kDa. The theoretical mass of rSarA, determined using the rSarA sequence, was found to be ~15.78 kDa. Taken together, we suggest the formation of rSarA homodimers in solution containing no TX-100. Our gel filtration chromatography did not show a peak corresponding to the complex formed between rSarA and TX-100 micelles. The absence of complex-specfic peak indicates either the formation of no complex or the dissociation of weak complex upon dilution in the column.

**Fig 4:**
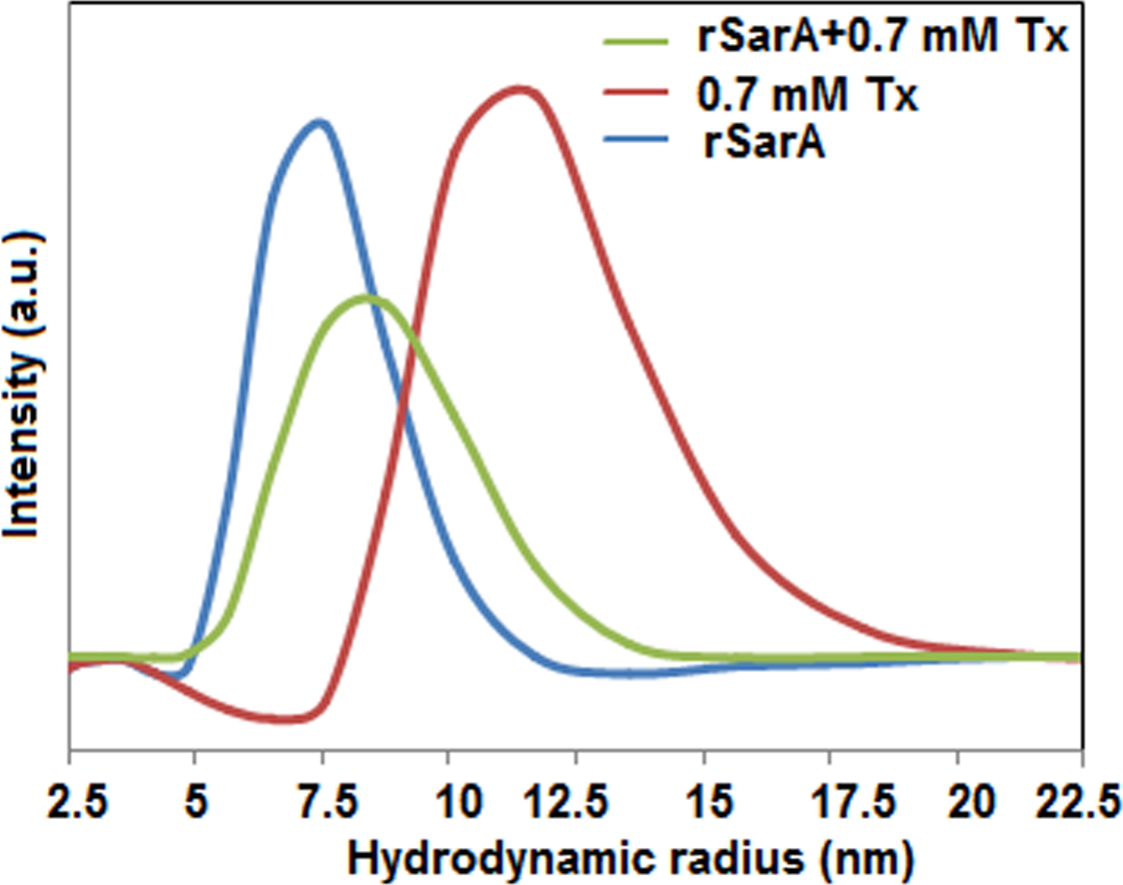
Shape of rSarA. Dynamic light scattering of rSarA, 0.7 mM TX-100, and rSarA plus 0.7 mM TX-100 (Tx). A set of scattering data are shown here.

Sizes of many proteins and their complexes with detergents have been determined using dynamic light scattering (DLS), an extremely sensitive probe [8, 43, 48–51]. To test if the dimeric rSarA truly forms complexes with TX-100, we have also measured the sizes of rSarA in the presence of 0 and 0.7 mM TX-100 using separate DLS experiments. The DLS estimation of the buffer containing 0.7 mM TX-100 has also been carried out for comparison. Fig 4 shows that the apparent hydrodynamic radii of TX-100-untreated rSarA, TX-100 and TX-100-equilibrated rSarA are ~7.53, ~11.7, and ~8.72 nm, respectively. The increase of size of rSarA in the presence of TX-100 might be due to the formation of complex between TX-100 micelle and rSarA. As the size of the complex is intermediate to those of rSarA and TX-100, there could be a relatively weaker interaction between rSarA and TX-100.

### Effects of TX-100 on the DNA binding activity of C9W

**Fig 5:**
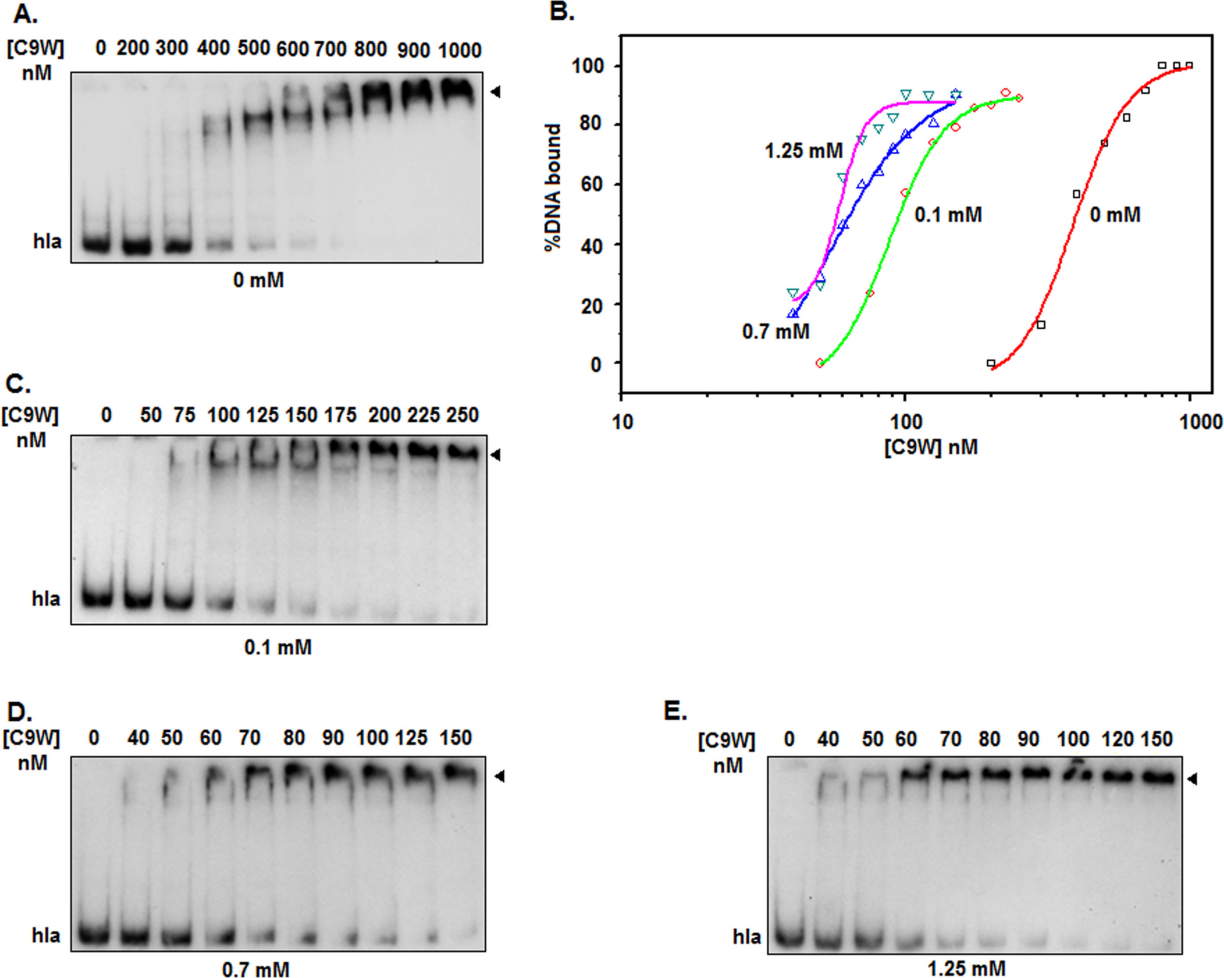
DNA binding activity of C9W at 0–1.25 mM TX-100. (A) Autoradiogram of the gel shift assay shows the equilibrium binding of C9W to the ^32^P-labeled hla DNA in the presence of 0 mM (A), 0.1 mM (C), 0.7 mM (D), and 1.25 mM TX-100 (E). Arrowhead represents the C9W-hla DNA complex. All of the assays are carried out at least three times. One set of autoradiograms are presented here. (B) DNA binding activity of C9W. The extents of C9W bound to hla DNA in the presence of denoted concentrations of TX-100 are estimated (from the data of the above autoradiograms) and plotted against the corresponding C9W concentrations.

Structures and functions of numerous proteins have been investigated by the intrinsic Trp fluorescence spectroscopy, a popular biophysical probe [46]. The absence of Trp residue in rSarA [9] has, however, restricted us to study this protein by intrinsic Trp fluorescence spectroscopy. To elaborately determine the effects of TX-100 on SarA, we have, therefore, constructed and purified C9W, a rSarA mutant with a Cys to Trp substitution at position 9. The lone Cys residue in SarA is the site where phosphorylation-dephosphorylation takes place [22, 23]. The phosphorylated SarA appears to enhance the affinity of SarA to some promoter DNAs. Previously, a SarA mutant with the Cys to Ala substitution, however, retained adequate DNA binding activity *in vitro* [12]. To check whether C9W has possessed any DNA binding activity, we have performed a gel shift assay using ^32^P-labeled hla DNA and varying concentrations of this mutant. The autoradiogram shows the effective binding of C9W with the labelled hla DNA (Fig 5A). Further analysis of the scanned data from the autoradiogram reveals that the hla DNA binding affinity of C9W is almost similar to that of rSarA (Fig 5B and Table 1).

To verify whether TX-100 also can similarly enhance the DNA binding activity of C9W, we have performed gel shift assays using the ^32^P-labeled hla DNA and C9W in the presence of 0.1, 0.7 and 1.25 mM TX-100.The complete binding of C9W to hla DNA also occurs at lower C9W concentration in the presence of TX-100 than in its absence (Fig 5C, 5D and 5E). The *K*_d_ values are calculated from the resulting plots of the percent DNA bound versus the C9W concentrations (Fig 5B and Table 1). The data together show that the hla DNA binding affinity of C9W in the presence of either concentration of TX-100 is considerably higher than that of same protein in the absence of TX-100 (all *p* values less than 0.05).

### Effects of TX-100 on the secondary and tertiary structures of C9W

**Fig 6:**
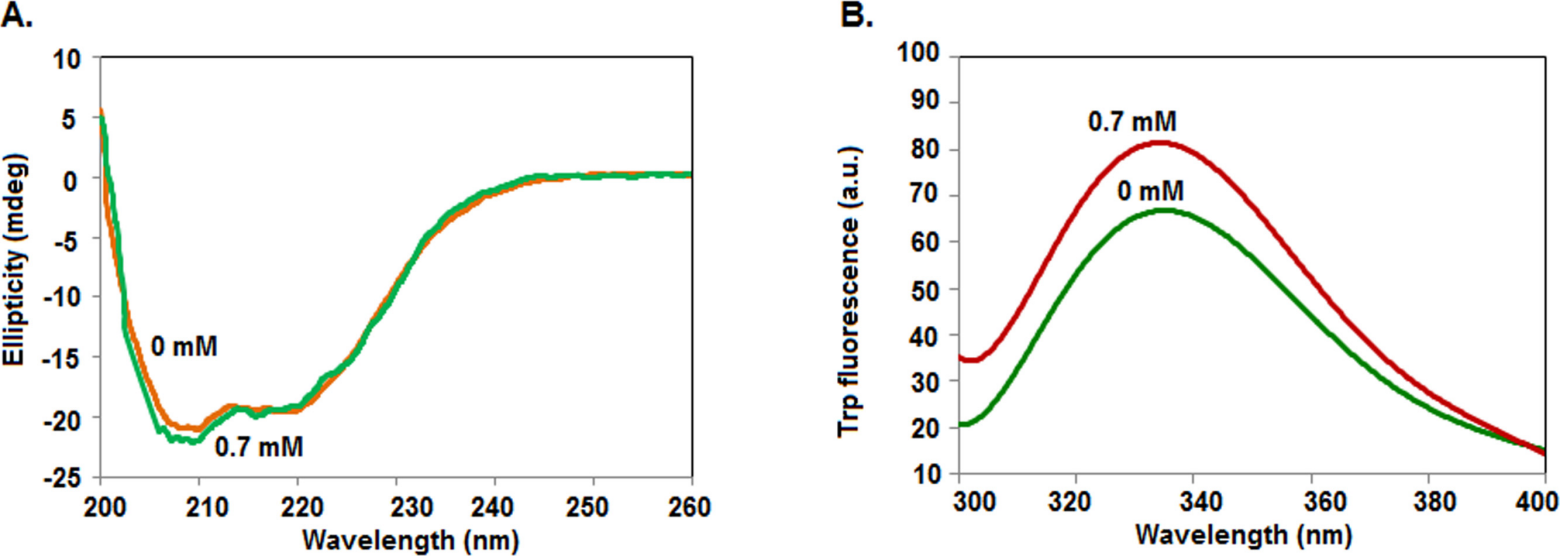
Secondary and tertiary structures of C9W. Far-UV CD (A) and intrinsic Trp fluorescence (B) spectra of C9W in the presence of indicated concentrations of TX-100. One set of spectra are presented here.

To check whether TX-100 also modifies the structure of C9W, we have separately recorded its far-UV CD and intrinsic Trp fluorescence spectra in the presence of 0 and 0.7 mM TX-100. Fig 6A reveals that the far-UV CD spectra of C9W in the presence of 0 and 0.7 mM TX-100 are not completely identical. Both spectra possess peaks at ~208 and ~220 nm, indicating the presence of α-helix in C9W at 0 mM and 0.7 mM TX-100. Additional analysis of the spectra shows the varying amounts of different secondary structural elements (including α-helix) in rSarA in the presence and absence of TX-100 (S2 Table).

To determine the effects of TX-100 on the tertiary structure of C9W, we have recorded the intrinsic Trp fluorescence spectra of this protein in the buffers containing 0 and 0.7 mM TX-100. Fig 6B shows a relatively higher fluorescence intensity of C9W in the presence of TX-100. In addition, the wavelength of emission maximum (λ_max_) values of the Trp fluorescence spectra of C9W in the absence and presence of TX-100 are 336 and 333 nm, respectively. The data together suggest a TX-100-induced structural alteration of C9W that further buries its Trp residue.

### Interaction between C9W and TX-100

**Fig 7:**
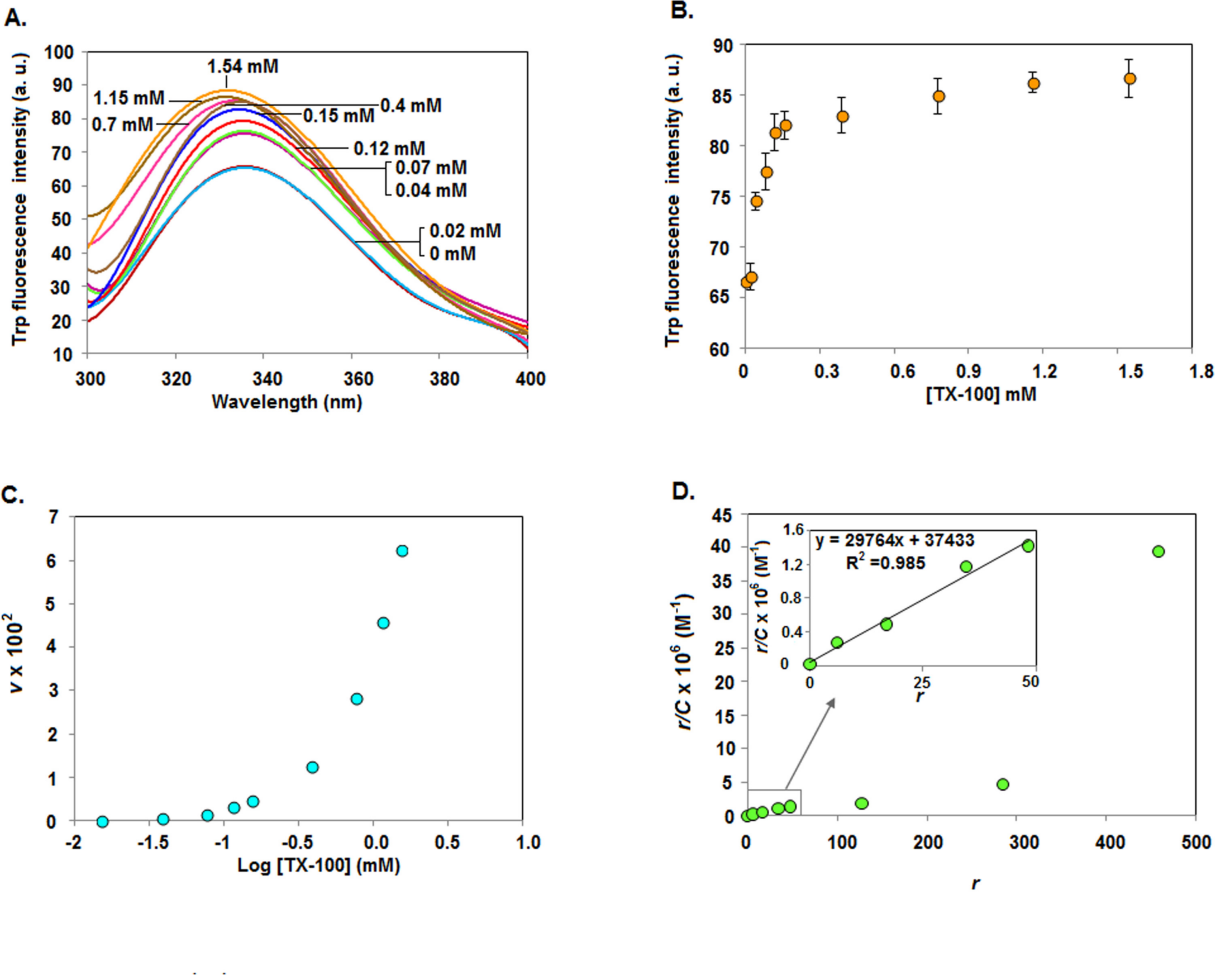
Binding of TX-100 to C9W. (A) The intrinsic Trp fluorescence spectra of C9W in the presence of 0–1.54 mM TX-100 (Tx). A set of spectra are shown here. (B) The Trp fluorescence intensity values of C9W at 336 nm, extracted from the fluorescence spectra in panel A, are plotted against the related TX-100 concentrations. (C) The average number of TX-100 molecule bound per C9W molecule (ν), determined using the Trp fluorescence intensity values stated in panel A and a standard equation [27], are plotted against the total concentration of added TX-100. (D) Scatchard plot showing the interaction between TX-100 and C9W has been developed as described in Materials and Methods. The inset plot, developed using the indicated values, is used to determine the C9W binding constant of TX-100.

Our spectroscopic and radioactive investigations indicate the interaction between rSarA/C9W and TX-100. To understand the nature of such interaction precisely, we have also recorded the intrinsic Trp fluorescence spectra of C9W in the presence of varying concentrations of TX-100 (Fig 7A). There is the gradual increase of the Trp fluorescence intensity of C9W when the concentrations of TX-100 have been raised from ~0 to 0.154 mM (Fig 7B). Thereafter, the fluorescence intensity values of C9W are not increased notably upon further increasing the TX-100 concentration to 1.54 mM. The λ_max_ values of C9W are decreased from 336 to 333 nm when the TX-100 concentration has been enhanced from 0 to 1.54 mM. Using the fluorescence intensity values, the amounts of TX-100 bound by C9W are determined and plotted against the TX-100 concentrations (Fig 7C). The resulting curve shows a slow rise at the TX-100 concentrations of ~0 to 0.154 mM, indicating that the binding of TX-100 to C9W has followed a non-cooperative mechanism at the pre-micellar concentrations of this surfactant. The binding curve, however, shows a steep rise at ~0.39–1.54 mM TX-100, suggesting that the binding of TX-100 to C9W is cooperative in nature at the micellar and post-micellar concentrations of this surfactant.

To determine the binding affinity of TX-100 to C9W, a Scatchard plot [47] was developed (Fig 7D) using the Trp fluorescence intensity values (Fig 7B) by a standard procedure as stated in Materials and Methods. The Scatchard plot appears to be non-linear, further indicating that the binding of TX-100 to C9W is primarily cooperative in nature. Using the linear part of the Scatchard plot, the affinity constant of TX-100 with C9W has been determined and observed to be ~3 x 10^4^ M^-1^. Additional analysis with the binding constant shows that the number of TX-100 binding sites on C9W is about 1.3. The binding constants of TX-100 with several globular proteins were reported to be in the order of ~10^2^−10^6^ M^-1^ [27–28]. Taken together, TX-100 binds C9W with a moderate affinity.

## Discussion

The present investigations for the first time have provided some clues on the interaction between a non-ionic surfactant (such as TX-100) and a DNA binding virulence regulator like *S*. *aureus* SarA. We have demonstrated a substantial increase of the DNA binding activity of rSarA/C9W in the presence of TX-100. In addition, secondary and tertiary structures of rSarA/C9W in the solution containing TX-100 micelles have been altered to some extent. There was even the formation of a complex between TX-100 micelles and rSarA. TX-100 at pre-micellar concentrations bound to C9W by a non-cooperative mechanism. The surfactant at micellar and post-micellar concentrations has, however, exhibited cooperative binding toC9W. Many other globular proteins also possess altered structure and shape upon binding non-ionic surfactants including TX-100 [27–29, 43, 48–50].

Several proteins (including transcription regulator), like rSarA, have shown a higher biological activity in the solution containing TX-100 [29, 51–54]. Molecules of the non-ionic surfactant usually interact with proteins using their respective hydrophobic regions [24]. Such interaction may lead to the partial denaturation of protein as well. The SarA dimeric interface formed by its helices α1, α2, and α5 is the most hydrophobic and buried region in this molecule (S4 Fig). Expectedly, most amino acid residues in the SarA dimeric interface are non-polar and possess relatively less crystallographic *B*-values [12]. Conversely, the residues with the highest *B*-values form the β-hairpin (or winged region) in SarA, indicating that this is the most surface-exposed region in SarA (S4 Fig). Our previous partial proteolysis data have indicated that the helix α2-forming residues are completely buried, whereas, residues forming helices α1 and α5 are predominantly within the interior of SarA dimer [8]. On the other hand, the winged region is extremely susceptible to the proteolytic enzymes. Taken together, the dimeric interface of SarA is the most possible target of TX-100 molecules.

Our intrinsic Trp fluorescence (Fig 7) and DLS (Fig 4) studies indicate that the affinity of TX-100 to dimeric rSarA is not very strong. The hypothesis has been partly supported by the observation that the secondary structure, tertiary structure, and dimerization status of rSarA are not severely affected by this surfactant (Fig 3, Fig 6 and S3 Fig). Possibly, the small structural alterations of rSarA and C9W resulted due to the moderate interactions between TX-100 and the dimeric interfaces of these proteins have oriented their HTH motifs and β-hairpins in a manner that finally have increased their DNA binding activities. As the DNA binding specificity of rSarA was not changed in the presence of TX-100 (Fig 2), this surfactant could be employed in the various DNA binding studies of SarA, which may in turn reveal the regions and residues of SarA and its cognate DNA involved in their interaction.

Lipoteichoic acid (LTA), a key component in the cell wall of *S*. *aureus*, usually regulates the expression of various autolysins or peptidoglycan hydrolases in this bacterium [55]. Several studies have indicated that the exposure of *S*. *aureus* to TX-100 induces the removal of acylated LTA from this pathogen [30–34]. The release of LTA, therefore, induces autolysis of *S*. *aureus*, particularly at the inhibitory concentrations of TX-100. Interestingly, the productions of autolysins and LTA in *S*. *aureus* are differently regulated by SarA [56, 57]. An elegant proteomic investigation has demonstrated that TX-100 alters the expression of many *S*. *aureus* proteins including SarA and two SarA-regulated proteins, Rot and IsaA [34]. Expression of Rot was substantially suppressed, whereas, that of IsaA was significantly enhanced in the presence of TX-100. Earlier studies have shown that SarA induces and represses the synthesis of IsaA and Rot, respectively [58, 59]. Currently, it is not clear whether the altered expression of Rot and IsaA have been occurred both due to the enhanced production and the structural alteration of SarA in the presence of TX-100.

## Conclusions

Our *in vitro* probes have indicated that the binding of TX-100 molecules to rSarA has not only considerably enhanced its DNA binding affinity but also altered its structure to some extent. TX-100 also has similarly modulated the DNA binding activity and structure of C9W, a Trp carrying variant of rSarA. TX-100 appears to bind rSarA or C9W with a moderate affinity. The information could be useful to determine the structure of SarA-DNA complex in the future.

## Supporting Information

S1 Fig Purification of C9W. Different protein containing fractions, collected from the affinity chromatography of SAU1336 cell extract, are analyzed by a SDS-13.5% PAGE. The uninduced, pellet, supernatant, flow-through, wash, and elution fractions are loaded in the lanes U, P, S, F, W, and E, respectively. The marker proteins are loaded in the lane M. Masses of different marker proteins (in kDa) are mentioned at the right side of gel.
(TIF)

S2 Fig Gel shift assay in the presence of 0 and 0.7 mM TX-100. Autoradiograms show the equilibrium binding of rSarA to the ^32^P-labeled spa DNA in the absence (A) and presence (B) of TX-100. Arrowhead indicates the rSarA-spa DNA complex. One set of autoradiograms are shown here. (C) rSarA DNA binding affinity. The amounts of rSarA bound to spa DNA in the presence /absence of 0.7 mM TX-100 are determined (from the autoradiograms mentioned above) and plotted against the corresponding rSarA concentrations.
(TIF)

S3 Fig Oligomeric status of rSarA. (A) Glutaraldehyde (GCHO)-mediated crosslinking of rSarA in the presence (+) /absence (-) of 0.7 mM TX-100 (Tx). Proteins treated and untreated with GCHO are analyzed by SDS-13.5% PAGE. The marker proteins are loaded in the lane M. Masses of different marker proteins (in kDa) are mentioned at the right side of gel. (B) Gel filtration chromatography of rSarA in the presence (+) /absence (-) of 0.7 mM TX-100 (Tx).
(TIF)

S4 Fig A three-dimensional structure of dimeric SarA. The ribbon structure of dimeric SarA [12] was developed by PyMol (www.pymol.org) on the basis of the crystallographic *B*-values of the composed residues. The α-helices and β-hairpin of one SarA monomer are indicated. The SarA regions represented by blue and red colors denote the most buried and surface-exposed regions of this molecule, respectively. The SarA regions denoted by other colors indicate varying levels of exposure to surface.
(TIF)

S1 Table Oligonucleotides used in the study. (DOCX)

S2 Table Secondary structural elements in rSarA and C9W. (DOCX)

## References

[1] Cheung AL, Bayer AS, Zhang G, Gresham H, Xiong YQ. Regulation of virulence determinants *in vitro* and *in vivo* in *Staphylococcus aureus*. FEMS Immunol Med Microbiol. 2004; 40: 1–9. 14734180 10.1016/S0928-8244(03)00309-2

[2] Bronner S, Monteil H, Prévost G. Regulation of virulence determinants in *Staphylococcus aureus*: complexity and applications. FEMS Microbiol Rev. 2004; 28: 183–200. 15109784 10.1016/j.femsre.2003.09.003

[3] Plata K, Rosato AE, Wegrzyn G. *Staphylococcus aureus* as an infectious agent: overview of biochemistry and molecular genetics of its pathogenicity. Acta Biochim Pol. 2009; 56: 597–612. 20011685

[4] Otto M. Basis of virulence in community-associated methicillin-resistant *Staphylococcus aureus*. Annu Rev Microbiol. 2010; 64:143–62. 10.1146/annurev.micro.112408.134309 20825344

[5] Cue D, Lei MG, Lee CY. Genetic regulation of the intercellular adhesion locus in staphylococci. Front Cell Infect Microbiol. 2012; 2: 38 10.3389/fcimb.2012.00038 23061050 PMC3459252

[6] Arya R, Princy SA. An insight into pleiotropic regulators Agr and Sar: molecular probes paving the new way for antivirulent therapy. Future Microbiol. 2013; 8: 1339–53. 10.2217/fmb.13.92 24059923

[7] Rechtin TM, Gillaspy AF, Schumacher MA, Brennan RG, Smeltzer MS, Hurlburt BK. Characterization of the SarA virulence gene regulator of *Staphylococcus aureus*. Mol Microbiol. 1999; 33: 307–316. 10411747 10.1046/j.1365-2958.1999.01474.x

[8] Mahapa A, Mandal S, Biswas A, Jana B, Polley S, Sau S. Chemical and thermal unfolding of a global Staphylococcal virulence regulator with a flexible C-terminal end. PLoS One. 2015; 10:e0122168 10.1371/journal.pone.0122168 25822635 PMC4379015

[9] Cheung AL, Nishina KA, Trotonda MP, Tamber S. The SarA protein family of *Staphylococcus aureus*. Int J Biochem Cell Biol. 2008; 40: 355–61. 18083623 10.1016/j.biocel.2007.10.032 PMC2274939

[10] Liu Y, Manna AC, Li R, Martin WE, Murphy RC, Cheung AL, et al Crystal structure of the SarR protein from *Staphylococcus aureus*. Proc Natl Acad Sci USA. 2001; 98: 6877–6882. 11381122 10.1073/pnas.121013398 PMC34446

[11] Li R, Manna AC, Dai S, Cheung AL, Zhang G. Crystal structure of the SarS protein from *Staphylococcus aureus*. J Bacteriol. 2003; 185: 4219–25. 12837797 10.1128/JB.185.14.4219-4225.2003 PMC164878

[12] Liu Y, Manna AC, Pan CH, Kriksunov IA, Thiel DJ, et al Structural and function analyses of the global regulatory protein SarA from *Staphylococcus aureus*. Proc Natl Acad Sci USA. 2006; 103: 2392–7. 16455801 10.1073/pnas.0510439103 PMC1413715

[13] Chen PR, Bae T, Williams WA, Duguid EM, Rice PA, Schneewind O, et al An oxidation-sensing mechanism is used by the global regulator MgrA in *Staphylococcus aureus*. Nat Chem Biol. 2006; 2: 591–5. 16980961 10.1038/nchembio820

[14] Poor CB, Chen PR, Duguid E, Rice PA, He C. Crystal structures of the reduced, sulfenic acid, and mixed disulfide forms of SarZ, a redox active global regulator in *Staphylococcus aureus*. J Biol Chem. 2009; 284: 23517–24. 10.1074/jbc.M109.015826 19586910 PMC2749125

[15] Zhu Y, Fan X, Zhang X, Jiang X, Niu L, Teng M, et al, Structure of Rot, a global regulator of virulence genes in *Staphylococcus aureus*. Acta Crystallogr D Biol Crystallogr. 2014; 70: 2467–76. 10.1107/S1399004714015326 25195759

[16] Ballal A, Manna AC. Regulation of superoxide dismutase (*sod*) genes by SarA in *Staphylococcus aureus*. J Bacteriol. 2009; 191: 3301–10. 10.1128/JB.01496-08 19286803 PMC2687179

[17] Ballal A, Manna AC. Control of thioredoxin reductase gene (*trxB*) transcription by SarA in *Staphylococcus aureus*. J Bacteriol. 2010; 192: 336–45. 10.1128/JB.01202-09 19854896 PMC2798248

[18] Dunman PM, Murphy E, Haney S, Palacios D, Tucker-Kellogg G, WU S, et al Transcription profiling-based identification of *Staphylococcus aureus* genes regulated by the *agr* and/or *sar*A loci. J Bacteriol. 2001; 183:7341–53. 11717293 10.1128/JB.183.24.7341-7353.2001 PMC95583

[19] Morrison JM, Anderson KL, Beenken KE, Smeltzer MS, Dunman PM. The staphylococcal accessory regulator, SarA, is an RNA-binding protein that modulates the mRNA turnover properties of late-exponential and stationary phase *Staphylococcus aureus* cells. Front Cell Infect Microbiol. 2012; 2: 26 10.3389/fcimb.2012.00026 22919618 PMC3417590

[20] Gordon CP, Williams P, Chan WC. Attenuating *Staphylococcus aureus* virulence gene regulation: a medicinal chemistry perspective. J Med Chem. 2013; 56: 1389–404. 10.1021/jm3014635 23294220 PMC3585718

[21] Fujimoto DF, Higginbotham RH, Sterba KM, Maleki SJ, Segall AM, Smeltzer SM, et al *Staphylococcus aureus* SarA is a regulatory protein responsive to redox and pH that can support bacteriophage lambda integrase-mediated excision/recombination. Mol Microbiol. 2009; 74:1445–58. 10.1111/j.1365-2958.2009.06942.x 19919677 PMC2879156

[22] Didier JP, Cozzone AJ, Duclos B. Phosphorylation of the virulence regulator SarA modulates its ability to bind DNA in *Staphylococcus aureus*. FEMS Microbiol Lett. 2010; 306: 30–6. 10.1111/j.1574-6968.2010.01930.x 20337713

[23] Sun F, Ding Y, Ji Q, Liang Z, Deng X, Wong CC, et al, Protein cysteine phosphorylation of SarA/MgrA family transcriptional regulators mediates bacterial virulence and antibiotic resistance. Proc Natl Acad Sci USA. 2012; 109: 15461–6. 22927394 10.1073/pnas.1205952109 PMC3458358

[24] Linke D. Detergents: an overview. Methods Enzymol. 2009; 463: 603–17. 10.1016/S0076-6879(09)63034-2 19892194

[25] Dimroth P, Thomer A. Solubilization and reconstitution of the Na(+)-dependent citrate carrier of *Klebsiella pneumoniae*. J Biol Chem. 1990; 265: 7721–4. 2186025

[26] Karbarz MJ, Six DA, Raetz CR. Purification and characterization of the lipid A 1 phosphatase LpxE of *Rhizobium leguminosarum*. J Biol Chem. 2009; 284: 414–25. 10.1074/jbc.M808390200 18984595 PMC2610509

[27] De S, Girigoswami A, Das S. Fluorescence probing of albumin-surfactant interaction. J Colloid Interface Sci. 2005; 285: 562–73. 15837473 10.1016/j.jcis.2004.12.022

[28] Singh SK, Kishore N. Thermodynamic insights into the binding of Triton X-100 to globular proteins: a calorimetric and spectroscopic investigation. J Phys Chem B. 2006; 110: 9728–37. 16686525 10.1021/jp0608426

[29] Weng L, Kohara M, Wakita T, Shimotohno K, Toyoda T. Detergent-induced activation of the hepatitis C virus genotype 1b RNA polymerase. Gene. 2012; 496: 79–87. 10.1016/j.gene.2012.01.044 22306265

[30] Raychaudhuri D, Chatterjee AN. Use of resistant mutants to study the interaction of triton X-100 with *Staphylococcus aureus*. J Bacteriol. 1985; 164:1337–49. 2866176 10.1128/jb.164.3.1337-1349.1985 PMC219335

[31] Komatsuzawa H, Suzuki J, Sugai M, Miyake Y, Suginaka H. The effect of Triton X 100 on the in-vitro susceptibility of methicillin-resistant *Staphylococcus aureus* to oxacillin. J Antimicrob Chemother.1994; 34: 885–97. 7730232 10.1093/jac/34.6.885

[32] Komatsuzawa H, Sugai M, Shirai C, Suzuki J, Hiramatsu K, Suginaka H. Triton X-100 alters the resistance level of methicillin-resistant *Staphylococcus aureus* to oxacillin. FEMS Microbiol Lett. 1995; 134: 209–12. 8586269 10.1111/j.1574-6968.1995.tb07939.x

[33] Suzuki J, Komatsuzawa H, Sugai M, Ohta K, Kozai K, Nagasaka N, et al, Effects of various types of Triton X on the susceptibilities of methicillin-resistant staphylococci to oxacillin. FEMS Microbiol Lett. 1997; 153: 327–31. 9271859 10.1111/j.1574-6968.1997.tb12592.x

[34] Cordwell SJ, Larsen MR, Cole RT, Walsh BJ. Comparative proteomics of *Staphylococcus aureus* and the response of methicillin-resistant and methicillin-sensitive strains to Triton X-100. Microbiology. 2002; 148: 2765–81. 12213923 10.1099/00221287-148-9-2765

[35] Ausubel FM, Brent R, Kingston RE, Moore DD, Seidman JG, Smith JA, et al, Current Protocols in Molecular Biology. John Wiley & Sons, Inc., USA 1998.

[36] Chien Y, Manna AC, Projan SJ, Cheung AL. SarA, a global regulator of virulence determinants in *Staphylococcus aureus*, binds to a conserved motif essential for *sar*-dependent gene regulation. J Biol Chem. 1999; 274: 37169–76. 10601279 10.1074/jbc.274.52.37169

[37] Sambrook J, Russell DW. Molecular Cloning: A Laboratory Manual. 3rd edn. Cold Spring Harbor Laboratory Press, Plainview, NY 2001.

[38] Jana B, Bandhu A, Mondal R, Biswas A, Sau K, Sau S. Domain structure and denaturation of a dimeric Mip-like peptidyl-prolyl cis-trans isomerase from *Escherichia coli*. Biochemistry. 2012; 51: 1223–37. 10.1021/bi2015037 22263615

[39] Biswas A, Mandal S, Sau S. The N-terminal domain of the repressor of *Staphylococcus aureus* phage Φ11 possesses an unusual dimerization ability and DNA binding affinity. PLoS One.2014; 9: e95012 10.1371/journal.pone.0095012 24747758 PMC3991615

[40] Liu H, Naismith JH. An efficient one-step site-directed deletion, insertion, single and multiple-site plasmid mutagenesis protocol. BMC Biotechnol. 2008; 8:91 10.1186/1472-6750-8-91 19055817 PMC2629768

[41] Bandhu A, Ganguly T, Jana B, Mondal R, Sau S. Regions and residues of an asymmetric operator DNA interacting with the monomeric repressor of temperate mycobacteriophage L1. Biochemistry. 2010; 49: 4235–43. 10.1021/bi9020956 20377203

[42] Ganguly T, Das M, Bandhu A, Chanda PK, Jana B, Sau A. Physicochemical properties and distinct DNA binding capacity of the repressor of temperate *Staphylococcus aureus* phage phi11. FEBS J. 2009; 276: 1975–85. 10.1111/j.1742-4658.2009.06924.x 19250317

[43] Biswas H, Chattopadhyaya R. Thermal, chemical and pH induced unfolding of turmeric root lectin: modes of denaturation. PLoS One. 2014; 9: e103579 10.1371/journal.pone.0103579 25140525 PMC4139268

[44] Creighton TE. Protein Structure: A Practical Approach, 2nd ed., IRL Press at Oxford University Press, New York 1997.

[45] Bohm G, Muhr R, Jaenicke R. Quantitative analysis of protein far UV circular dichroism spectra by neural networks. Protein Eng. 1992; 5: 191–195. 1409538 10.1093/protein/5.3.191

[46] Lakowicz JR. Principles of fluorescence spectroscopy, 2nd ed., Kluwer Academic/Plenum, New York 1999.

[47] Scatchard G. The attraction of proteins for small molecules and ions. Ann NY Acad Sci. 1949; 51: 660–665.

[48] Tatkiewicz W, Elizondo E, Moreno E, Díez-Gil C, Ventosa N, Veciana J, Ratera I. Methods for characterization of protein aggregates. Methods Mol Biol. 2015; 1258: 387–401. 10.1007/978-1-4939-2205-5_22 25447877

[49] Ruiz CC, Hierrezuelo JM, Aguiar J, Peula-García JM. Physicochemical Studies on the Interaction between N-Decanoyl-N-methylglucamide and Bovine Serum Albumin. Biomacromolecules. 2007; 8: 2497–503. 17630693 10.1021/bm0704121

[50] Ruiz CC, Molina-Bolívar JA. Characterization of mixed non-ionic surfactants n-octyl-β-D-thioglucoside and octaethylene-glycol monododecyl ether: micellization and microstructure. J Colloid Interface Sci. 2011; 361: 178–85. 10.1016/j.jcis.2011.05.019 21641607

[51] Zalucki YM, Dhulipala V, Shafer WM. Dueling regulatory properties of a transcriptional activator (MtrA) and repressor (MtrR) that control efflux pump gene expression in *Neisseria gonorrhoeae*. MBio. 2012; 3: e00446–12. 10.1128/mBio.00446-12 23221802 PMC3517864

[52] Choi GH, Jo MN, Kim JM, Kim CJ, Kim KT, Palik HD, et al, Purification and characterization of heat-tolerant protease produced by *Bacillus polyfermenticus* SCD. J Microbiol Biotechnol. 2013; 23: 1554–9. 23949331 10.4014/jmb1306.06073

[53] Grover N, Paskaleva EE, Mehta KK, Dordick JS, Kane RS. Growth inhibition of *Mycobacterium smegmatis* by mycobacteriophage-derived enzymes. Enzyme Microb Technol. 2014; 63:1–6. 10.1016/j.enzmictec.2014.04.018 25039052

[54] Juntachai W, Oura T, Kajiwara S. Purification and characterization of a secretory lipolytic enzyme, MgLIP2, from *Malassezia globosa*. Microbiology. 2011; 157: 3492–9. 10.1099/mic.0.054528-0 22016565

[55] Holtje JV, Tomasz A. Biological effects of lipoteichoic acids. J Bacteriol. 1975; 124: 1023–7. 241741 10.1128/jb.124.2.1023-1027.1975 PMC235993

[56] Fujimoto DF, Bayles KW. Opposing roles of the *Staphylococcus aureus* virulence regulators, Agr and Sar, in Triton X-100- and penicillin-induced autolysis. J Bacteriol. 1998; 180: 3724–6. 9658022 10.1128/jb.180.14.3724-3726.1998 PMC107347

[57] Dunman PM, Murphy E, Haney S, Palacios D, Tucker-Kellogg G, Wu S, et al, Transcription profiling-based identification of *Staphylococcus aureus* genes regulated by the *agr* and/or *sarA* loci. J Bacteriol. 2001; 183: 7341–53. 11717293 10.1128/JB.183.24.7341-7353.2001 PMC95583

[58] Ziebandt AK, Weber H, Rudolph J, Schmid R, Höper D, Engelmann S, et al, Extracellular proteins of *Staphylococcus aureus* and the role of SarA and sigma B. Proteomics. 2001; 1: 480–93. 11681202 10.1002/1615-9861(200104)1:4<480::AID-PROT480>3.0.CO;2-O

[59] Manna AC, Ray B. Regulation and characterization of *rot* transcription in *Staphylococcus aureus*. Microbiology. 2007; 153: 1538–45. 17464068 10.1099/mic.0.2006/004309-0

