# Supplementary figures and images for: A Surfactant-Induced Functional Modulation of a Global Virulence Regulator from Staphylococcus aureus
Source: PLoS One. 2016 Mar 18;11(3):e0151426. doi: 10.1371/journal.pone.0151426 (PMC4798592; doi:10.1371/journal.pone.0151426)

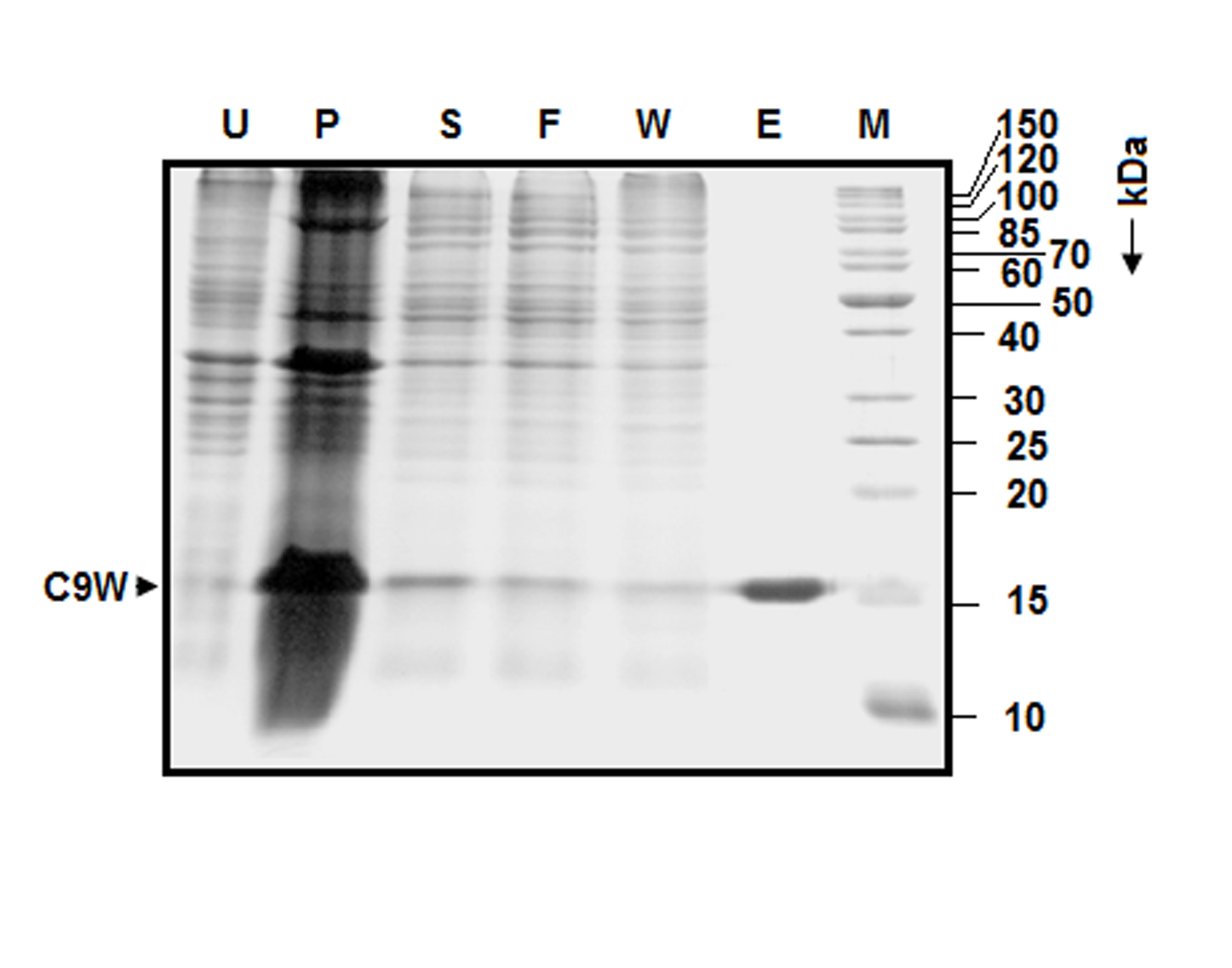

Supplement: S1 Fig — Different protein containing fractions, collected from the affinity chromatography of SAU1336 cell extract, are analyzed by a SDS-13.5% PAGE. The uninduced, pellet, supernatant, flow-through, wash, and elution fractions are loaded in the lanes U, P, S, F, W, and E, respectively. The marker proteins are loaded in the lane M. Masses of different marker proteins (in kDa) are mentioned at the right side of gel. (TIF) [file pone.0151426.s001.tif]

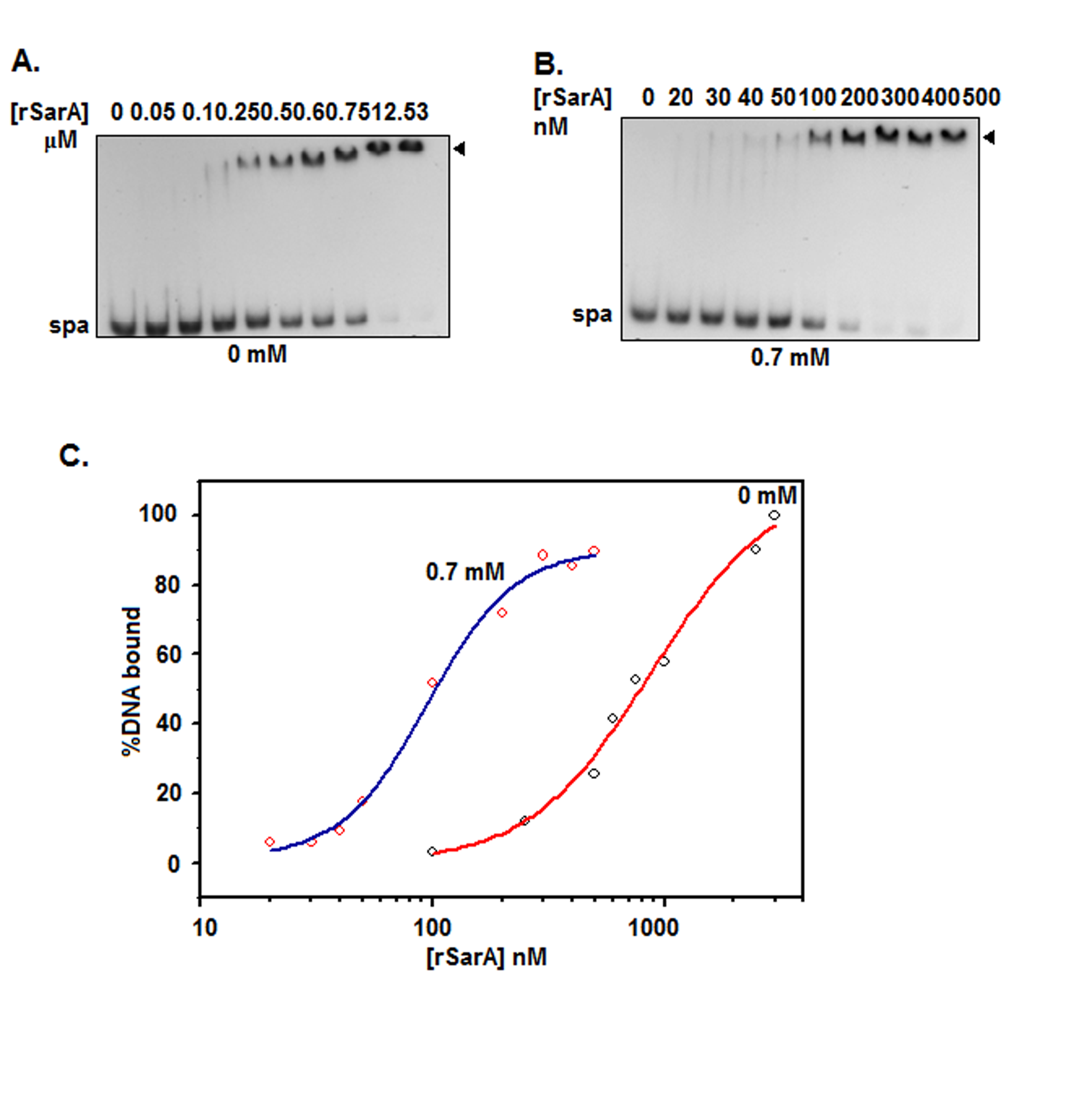

Supplement: S2 Fig — Autoradiograms show the equilibrium binding of rSarA to the 32P-labeled spa DNA in the absence (A) and presence (B) of TX-100. Arrowhead indicates the rSarA-spa DNA complex. One set of autoradiograms are shown here. (C) rSarA DNA binding affinity. The amounts of rSarA bound to spa DNA in the presence /absence of 0.7 mM TX-100 are determined (from the autoradiograms mentioned above) and plotted against the corresponding rSarA concentrations. (TIF) [file pone.0151426.s002.tif]

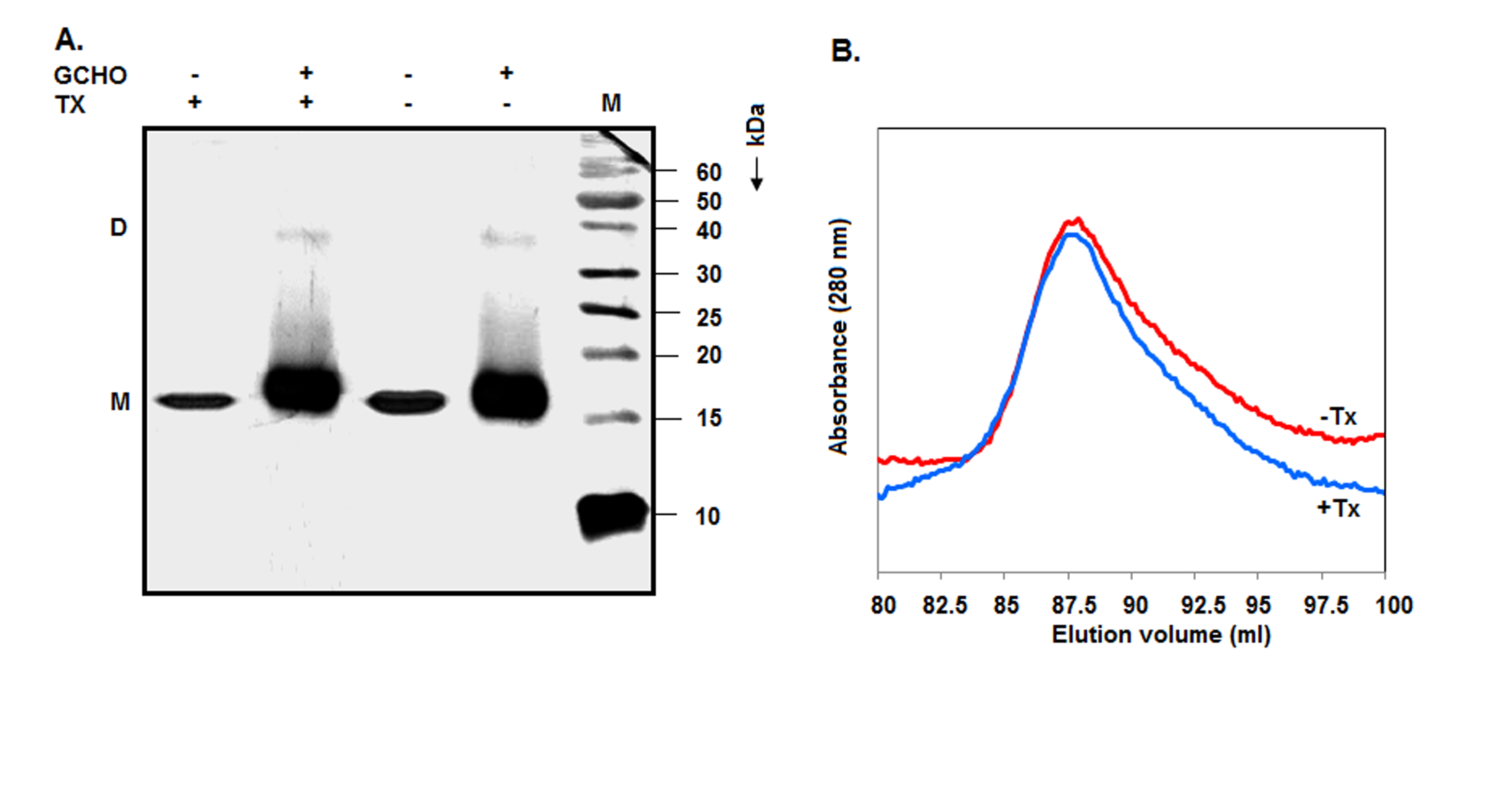

Supplement: S3 Fig — (A) Glutaraldehyde (GCHO)-mediated crosslinking of rSarA in the presence (+) /absence (-) of 0.7 mM TX-100 (Tx). Proteins treated and untreated with GCHO are analyzed by SDS-13.5% PAGE. The marker proteins are loaded in the lane M. Masses of different marker proteins (in kDa) are mentioned at the right side of gel. (B) Gel filtration chromatography of rSarA in the presence (+) /absence (-) of 0.7 mM TX-100 (Tx). (TIF) [file pone.0151426.s003.tif]

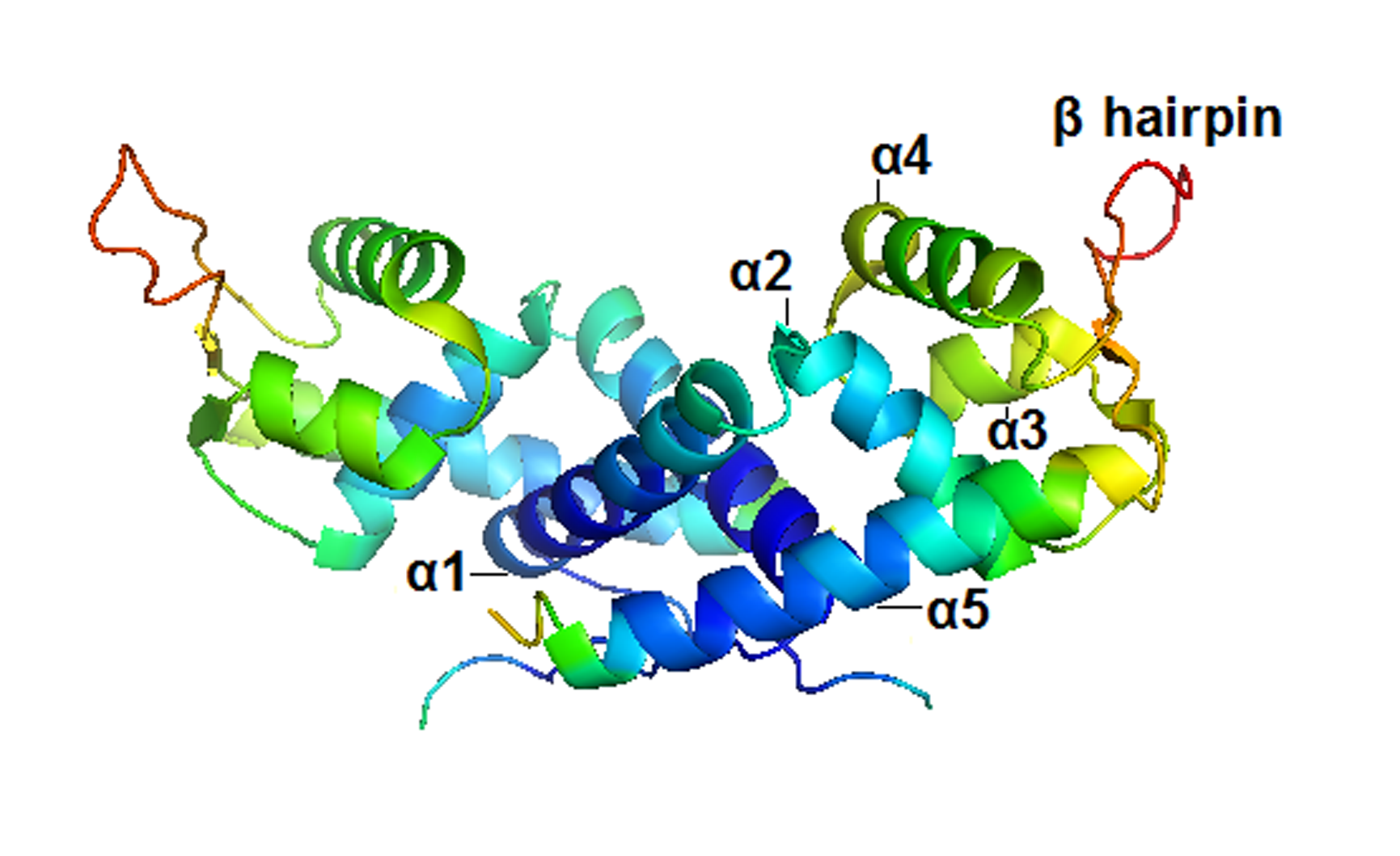

Supplement: S4 Fig — The ribbon structure of dimeric SarA [12] was developed by PyMol (www.pymol.org) on the basis of the crystallographic B-values of the composed residues. The α-helices and β-hairpin of one SarA monomer are indicated. The SarA regions represented by blue and red colors denote the most buried and surface-exposed regions of this molecule, respectively. The SarA regions denoted by other colors indicate varying levels of exposure to surface. (TIF) [file pone.0151426.s004.tif]
