# Supplementary material for: A Surfactant-Induced Functional Modulation of a Global Virulence Regulator from Staphylococcus aureus
Source: PLoS One. 2016 Mar 18;11(3):e0151426. doi: 10.1371/journal.pone.0151426 (PMC4798592; doi:10.1371/journal.pone.0151426)
Supplement: S1 Table — (DOCX) [file pone.0151426.s005.docx]

**S1 Table. Oligonucleotides used in the study**

| **Name of oligonucleotide** | **Sequence of oligonucleotide (5’-3’)** | **Reference or source** |
| --- | --- | --- |
| SarA1 | CATACCATGGCAATTACAAAAATCAATG | 8 |
| SarA2 | CATACTCGAGTAGTTCAATTTCGTTGTTTG | 8 |
| Hla1 | Acatagctaattttattg | 8 |
| Hla2 | ctattagatatttctatg | 8 |
| Spa1 | agtcatcataatataacg | This study |
| Spa2 | cgaaatagcgtgattttg | This study |
| SarAC9W1 | AAAATCAATGATTGGTTTGAGTTGTTA | This study |
| SarAC9W2 | TAACAACTCAAAC CAATCATTGATTTT | This study |
