# Supplementary material for: A Surfactant-Induced Functional Modulation of a Global Virulence Regulator from Staphylococcus aureus
Source: PLoS One. 2016 Mar 18;11(3):e0151426. doi: 10.1371/journal.pone.0151426 (PMC4798592; doi:10.1371/journal.pone.0151426)
Supplement: S2 Table — (DOCX) [file pone.0151426.s006.docx]

**S2 Table. Secondary structural elements in proteins^*^**

| **Name of secondary structure** | **Amount (%) of secondary structure in rSarA with** | | **Amount (%) of secondary structure in C9W with** | |
| --- | --- | --- | --- | --- |
|  | **0 mM TX-100** | **0.7 mM TX-100** | **0 mM TX-100** | **0.7 mM TX-100** |
| α-Helix | 75.7 | 88.2 | 51.3 | 52.8 |
| β-Strand (anti-parallel) | 2 | 0.7 | 5.1 | 4.9 |
| β-Strand (parallel) | 2.1 | 0.8 | 5.4 | 5.2 |
| β-Turn | 10.8 | 7.1 | 14.4 | 14.1 |
| Random coil | 9.4 | 3.2 | 23.8 | 23.0 |

^*^The amount of secondary structures in rSarA or C9W, pre-equilibrated with/without TX-100, were determined by analyzing the corresponding far-UV CD spectra (Figs. 3A and 6A) with CDNN [45].
